# Evaluation of Second-Level Inference in fMRI Analysis

**DOI:** 10.1155/2016/1068434

**Published:** 2015-12-27

**Authors:** Sanne P. Roels, Tom Loeys, Beatrijs Moerkerke

**Affiliations:** Department of Data Analysis, Ghent University, H. Dunantlaan 1, 9000 Ghent, Belgium

## Abstract

We investigate the impact of decisions in the second-level (i.e., over subjects) inferential process in functional magnetic resonance imaging on (1) the balance between false positives and false negatives and on (2) the data-analytical stability, both proxies for the reproducibility of results. Second-level analysis based on a mass univariate approach typically consists of 3 phases. First, one proceeds via a general linear model for a test image that consists of pooled information from different subjects. We evaluate models that take into account first-level (within-subjects) variability and models that do not take into account this variability. Second, one proceeds via inference based on parametrical assumptions or via permutation-based inference. Third, we evaluate 3 commonly used procedures to address the multiple testing problem: familywise error rate correction, False Discovery Rate (FDR) correction, and a two-step procedure with minimal cluster size. Based on a simulation study and real data we find that the two-step procedure with minimal cluster size results in most stable results, followed by the familywise error rate correction. The FDR results in most variable results, for both permutation-based inference and parametrical inference. Modeling the subject-specific variability yields a better balance between false positives and false negatives when using parametric inference.

## 1. Introduction

In cognitive neurosciences, functional Magnetic Resonance Imaging (fMRI) plays an important role to localize brain regions and to study interactions among those regions (resp., functional segregation and functional integration; see, e.g., [1]) The analysis of an fMRI time course in a single subject (first-level analysis) offers some insight into subject-specific brain functioning while group studies that aggregate results over individuals (second-level analysis) yield more generalizable results. In this paper, we focus on the mass univariate approach in which the brain is divided in small volume units or voxels, although alternatives exist (e.g., [2]). For each of these voxels, a general linear model (GLM) is used to model brain activation, at the first and the second level [3]. The activation is then judged at the voxel level, rather than based on topological features. The selection of activated voxels can be viewed as a sequence of different phases [4]. For first-level analyses, Carp [5] demonstrated the large variation in the choices made in each of these different phases which impacts results. In second-level analyses, to a lesser extent, different combinations of choices are possible too. We consider the following phases in the analysis of group studies: (1) aggregation of data over subjects, (2) inference, and (3) correction for multiple testing.

In two commonly used software programs to analyze fMRI data (i.e., SPM and FSL [5]), the expected activation in each voxel is modeled in a two-step approach [6]. In the first-level analysis, the evidence per subject is summarized in a linear contrast of the parameters, necessary to model the study design. These contrast images are then passed to the second-level analysis in which the evidence is weighted over subjects. To pool this information over subjects, one can either take into account subject-specific variability in constructing the voxelwise test statistics or only rely on the estimated contrasts and not take into account this subject-specific variability [7].

After pooling the data, one proceeds to the second phase, the inference phase. While parametric inference offers the advantage of closed-form null distributions that can be used to obtain *p* values, it depends on strong assumptions which are not easy to satisfy in practice [8] and have not been tested extensively [9]. An alternative is to use nonparametric methods such as permutation-based inference to create an empirical null distribution conditional on the observed sample [9–11].

Third, inference must be corrected for the huge multiple testing that is induced by the mass univariate approach in which simultaneously over 100.000 tests are performed. As Bennett et al. [12] and Lieberman and Cunningham [13] discuss, there was (and yet is) no golden standard to address the choice for multiple testing corrections. We consider three different multiple testing procedures: controlling the False Discovery Rate (FDR), controlling the familywise error rate (FWE), and an approach based on uncorrected testing combined with a minimal cluster size. While FDR [14, 15] and FWE control (see, e.g., [8]) have a strong theoretical background with a focus, respectively, on the proportion of false positives among all selected voxels and on the probability to observe at least one false positive, the third approach is purely empirical in nature [13].

These three corrections are designed to control the multiple testing problem at the voxel level. Other popular alternatives that focus on topological features such as cluster size (i.e., the size of a neighboring collection of voxels) or cluster height exist as well. In a recent study, Woo et al. [16] advocate against the use of cluster-based inference and demonstrate its problematic use when studies are sufficiently powered. By definition, it is cumbersome to interpret the findings resulting from “significant clusters” because these may not reflect a set of significant constituting voxels (see also [9]). On the other hand, the third approach [13] resembles cluster-based testing but instead of setting a threshold for cluster size based on cluster significance, a fixed prespecified threshold for the minimum cluster size is set. For completeness, we therefore also extend the third approach by choosing the threshold as in cluster-based inference. However, it is important to point out that we do not intend to investigate cluster-based testing which is fundamentally different from the approach taken here and relies on different topological assumptions. Instead, we focus on voxelwise testing (for an elaborate investigation of cluster-based testing, we refer to [4]).

The choices made in each of the 3 phases of a second-level analysis is crucial steps in the analysis of fMRI data and may consequently influence results. The use of such second-level analyses or group studies is widespread [6, 10, 17, 18] but the impact of varying procedures at the different phases has not yet been extensively validated. One can distinguish three different aspects in the evaluation of methods [4]: validity, reliability, and stability. The validity can be assessed by verifying whether the false positive rate is controlled at a predefined, nominal level. Further, the balance between type I errors (false positives) and type II errors (false negatives) has long been the main interest in the validation of testing procedures (e.g., [8]). One has also acknowledged the importance of investigating the reliability of methods (e.g., [19, 20]). The extent to which a method is reliable can be measured through the overlap between activated brain regions over repeated measures, for example, in test-retest settings.

The concept of data-analytical stability, originally developed in genetics [21], was recently introduced into the context of fMRI data analysis [4]. This measure allows us to quantify reproducibility of results through the variability on different measures, for example, the variance on the number of selected voxels over replications (either in simulation studies with a known ground truth or through subsampling of real data). Stable methods are characterized by a low variability on the number of selected voxels. Data-analytical stability is thus a useful additional criterion to distinguish between methods. In this paper, we assess the influence of different choices made in the three phases on the reproducibility of results. We hereby focus on the balance between false positives and false negatives and on the stability as measures for reproducibility.

In Section 2 we give a brief overview of the different techniques. Next, we describe the details and the results of our simulation study. In Section 4, we present the results and the details from the real data application. In Discussion, we summarize our findings and end with some recommendations for the practitioner.

## 2. Methods

In this section we provide an overview on the different inferential techniques that we will consider in the simulation study and real data example. First, we describe the methods for pooling the evidence over subjects in the mass univariate GLM approach for fMRI data at the second level. Next, we summarize different multiple testing strategies that are frequently exploited in the fMRI literature, such as approaches that control the familywise error rate, approaches for control of the False Discovery Rate, and a two-step procedure based on an uncorrected threshold but requiring a minimum cluster size. Finally, we discuss the construction of test statistics under the null hypothesis that rely on parametric assumptions versus nonparametric approaches.

### 2.1. Voxel-Based GLM Approach to Analyzing fMRI Data at the Group Level

Group-level inference typically proceeds via a two-step procedure [6]. In the first step, an analysis is conducted at the voxel level for each subject *m* separately (with *m* = 1,…, *M*), and an appropriate contrast of interest is constructed. In a second step, these contrast images are combined to weight evidence over the *M* subjects.

#### 2.1.1. First-Level Analysis

For each subject *m*, the BOLD signal is sampled on *T* time points in every voxel *v* (with *v* = 1,…, *V*) during an fMRI experiment. For every voxel *v*, a general linear model (GLM) is then used to relate the voxels' time course (i.e., the BOLD signal) **Y**_**v**_ = (*Y*_*v*1_,…, *Y*_*vt*_,…, *Y*_*vT*_) to the expected BOLD signal under brain activation in the experimental setup (the design matrix **X**) (see, e.g., [22–25]):

(1) $$\begin{array}{c} Y_{v}=X\beta_{v}+\varepsilon_{v}. \end{array}$$

The design matrix **X** is the product of a convolution of the stimulus onset function with a hemodynamic response function (HRF) (e.g., [26]). When fitting model (1), one needs to account for the residual correlation between consecutive time points. Let **A** *σ*_*ɛ*_^2^ represent the variance-covariance matrix of **ε**_*v*_ in model (1). To deal with the temporal correlation, a matrix Σ_**d**_ is typically constructed such that Σ_**d**_ **A**Σ_**d**_^**t**^ = **I** holds. If **A** and **X** are correctly specified, **β**_**v**_ can be unbiasedly estimated via a simple least squares approach. By relying on “decorrelated” or whitened outcome and predictor, that is, **Y** and **X** are premultiplied by Σ_**d**_^−1^, an unbiased estimator for the variance of the estimator for **β**_**v**_ is obtained (see, e.g., [3, 27, 28]). Testing for specific differences between the activation in conditions for voxel *v* is then possible by testing the appropriate contrasts of the elements of **β**_*v*_ with a contrast vector **c**, that is, test *H*_0_ : **c** **β**_*v*_ = 0.

#### 2.1.2. Second-Level Analysis

Next we focus on the group level analysis for a specific voxel *v* (*v* = 1,…, *V*). For ease of notation, we will drop the voxel index *v* in the text below. For the contrast of interest, let **b** = [*b*_1_,…,*b*_*M*_]^*t*^ denote ${\left[c{\hat{\beta }}_{1},\ldots ,c{\hat{\beta }}_{M}\right]}^{t}$, the estimated contrasts at the first level for subjects 1 to *M*. Obviously, those contrasts are not exactly known but estimated with some imprecision. Suppose for now that those contrasts are known and denoted by **c** **β**, then a GLM can be used to weight the group evidence (e.g., [18]):

(2) $$\begin{array}{c} c\beta =X_{M}\gamma +\eta , \end{array}$$

where **X**_**M**_ denotes the design matrix. In the simplest case where one is interested in knowing whether there is activation over all subjects, the design matrix **X**_**M**_ equals a simple column matrix consisting of *M* elements 1. Alternatively, in the presence of between-subjects conditions or groups (e.g., one wants to know whether the activation is different between males and females), **X**_**M**_ can take more complex forms with additional regressors. Furthermore **η** is the group error vector, with Var(**η**) = *σ*_*η*_^2^ **I**_**M**_ with **I**_**M**_ the identity matrix of dimension *M* and *σ*_*η*_^2^ the between-subject variance.

In practice however **c** **β** is unknown, and instead **b** is used as outcome:

(3) $$\begin{array}{c} b=X_{M}\gamma +\eta^{*}, \end{array}$$

with **η** ^*∗*^ = [*η*_1_^*∗*^,…,*η*_*M*_^*∗*^]^*t*^ and **η** ^*∗*^ ~ *N*(0, Σ_**η**_^*∗*^). Since **η** ^*∗*^ = **c** **β** − **b** + **η**, it follows that the variance-covariance matrix Σ_*η*_^*∗*^ consists of the sum of two parts:

(4) Ση∗=varMb+ση2IM,

(5) Ση∗=ΣM+ση2IM,

(6) Ση∗=σ12000⋮000σM2︸within-subject+ση2IM︸between-subject.

The first term in the right hand side of (4) is inherent to the uncertainty associated with the estimation of **c** **β**_**m**_, the within-subject variability, while the second term is related to the variability in the estimation of **γ**, that is, the between-subjects variance.

In the literature on multisubject fMRI data analysis, two ways of dealing Σ_*m*_ are frequently used. Below, we refer to these two approaches as the Ordinary Least Squares (OLS) approach and the Weighted Least Squares (WLS) approach, respectively.

*OLS: The Homoscedastic Case*. In the first case, described in Holmes and Friston [17], one assumes that within-subject variances do not differ over subjects and that the residual noise is homogeneous across all *M* subjects. Assume that *σ*_1_^2^ = ⋯ = *σ*_*M*_^2^ simplifies the form of Σ_*η*_^*∗*^ (in model (6)) to

(7) $$\begin{array}{c} \Sigma_{\eta *}=\sigma_{\text{ols}}^{2}I_{M}. \end{array}$$

This implies that the within- and between-subject variability cannot be disentangled.

Mumford and Nichols [18] demonstrate that **γ** in model (3) (p 1470, in (6)) can then be estimated as ${\hat{\gamma }}_{\text{ols}}=X_{m}^{-1}b$ while the residual error variance *σ*_ols_^2^ is estimated as ${\left(b-X_{m}\hat{\gamma }\right)}^{\prime }\left(b-X_{m}\hat{\gamma }\right)/(M-1)$. Hence, this simply amounts to solving the normal equations in the simple linear regression case and inference proceeds as usual under the GLM [28]. This is implemented in FSL [29] under O LS while in SPM [30] this is the standard implementation. In AFNI [31] this is implemented under 3dttest++ (see also [32]).

*WLS: Allowing for Heteroscedasticity*. The WLS approach, or more generally the Generalized Least Squares (GLS) approach, explicitly models the two components of the variance-covariance of **η** ^*∗*^ in (6):

(8) $$\begin{array}{c} \Sigma_{\eta }^{*}=\left[\begin{array}{ccc} \sigma_{1}^{2}+\sigma_{\eta }^{2} & 0 & 0\\ 0 & \vdots & 0\\ 0 & 0 & \sigma_{M}^{2}+\sigma_{\eta }^{2} \end{array}\right]. \end{array}$$

More specifically, a weighting matrix *W* is constructed such that more variable estimates *b*_*m*_ are down-weighted in the estimation of **γ**. In the special case where the design matrix **X**_**m**_ only consists of a column of 1's, the closed form expression for the estimator of **γ** equals [18]

(9) $$\begin{array}{c} {\hat{\gamma }}_{\text{wls}}=\overset{M}{\underset{m=M}{\sum }}\frac{b_{i}}{\sigma_{m}^{2}+\sigma_{\eta }^{2}}{\left(\;\sum_{m=1}^{M}\frac{1}{\sigma_{m}^{2}+\sigma_{\eta }^{2}}\;\right)}^{-1}. \end{array}$$

More generally, ${\hat{\gamma }}_{\text{wls}}$ equals

(10) $$\begin{array}{c} {\left(\;X_{m}^{t}\hat{W}X_{m}\;\right)}^{-1}X_{m}^{t}{\hat{W}}^{-1}b \end{array}$$

with **W** the weighting matrix:

(11) $$\begin{array}{c} W=\left[\begin{array}{ccc} \left(\sigma_{1}^{2}+\sigma_{\eta }^{2}\right) & 0 & 0\\ 0 & \vdots & 0\\ 0 & 0 & \left(\sigma_{M}^{2}+\sigma_{\eta }^{2}\right) \end{array}\right]. \end{array}$$

Inference for the variance components is more complex since no closed form solutions exist. Several (restricted) maximal likelihood approaches have been suggested in the literature (see, e.g., [32]). In practice, the within-subject variance is often set to the first-level variance estimates ([18], also in the FSL software package).

In FSL this is implemented under Flame1 while in AFNI this is implemented under 3dMEMA (see also [33]).

### 2.2. Dealing with the Multiple Testing Problem

It is well-known that the mass-univariate approach in which *V* (*V* > 100.000) voxels are tested simultaneously is faced with huge multiple testing problem, even at the second level. Indeed, if 100.000 tests for which *H*_0_ is true are conducted simultaneously, each at a significance level of *α* = 0.05, then, by chance alone, 5000 voxels will be declared active. Hence, the number of false positives (FP, see Table 1) becomes unacceptably high. While the interest lies in minimizing both the number of FPs and false negatives (FNs), multiple testing procedures aim to control FP rates (type I error rates).

#### 2.2.1. Familywise Error Rate (FWE)

The FWE is the probability that at least one FP occurs among all tests performed (see, e.g., [8]). In order to control this error rate, one needs the null distribution of the maximum statistic over the *V* test statistics: max⁡(*T*_*v*_). Indeed, assuming that the global null (i.e., the null hypothesis holds for all voxels) holds, we have that

(12) PFP>0 ∣ global  H0=P⋃v=1V Tv>u ∣ global  H0=Pmax⁡Tv>u ∣ global  H0.

Hence, when *u* is chosen such that this probability is lower or equal to *α*, the FWE is controlled at level *α*. In fMRI data analysis, the most commonly used approach to controlling the FWE is based on Random Field Theory (RFT, see, e.g., [34]). Relying on parametric assumptions, RFT allows a closed form approximation of the upper tail of the null distribution of the maximum statistic. Alternatively, nonparametric methods for inference such as permutation-based testing may be used. In the latter case. This will be discussed more extensively in Section 2.3.2.

Note that the expressions in (12) imply weak control of the FWE as control is only guaranteed under the assumption that the null is true for all voxels. Nichols and Hayasaka [8, Section 2.3] argue that in imaging this weak control of FWE also entails strong control, that is, control for any subset of null voxels. This is essential to localize individual significant voxels.

Further note that the classical Bonferroni correction, in which the observed *p* value is multiplied with the number of tests and compared with to *α*, can also be used to control the FWE. The underlying assumption of independence when using the Bonferroni correction implies very conservative results in the fMRI context however and makes the Bonferroni correction relatively useless. While corrections for dependence exist, these are seldom used in the analysis of neuroimaging data [8].

#### 2.2.2. False Discovery Rate (FDR)

FWE is a very stringent error rate and controlling it leads to conservative corrections. Given that one is willing to accept more FPs, provided that this number is small relative to the total number of selected voxels, one can rely on a different error measure, the False Discovery Rate (FDR). The FDR equals *E*(*Q*) with

(13) Q=#FP#selected  voxels=#FP#FP+#TPif  #  selected  voxels>00otherwise.

Genovese et al. [15] introduced a procedure to control the FDR in neuroimaging. Using the procedure of Benjamini and Hochberg [14], the FDR is considered at level *q* in the sense that

(14) $$\begin{array}{c} E\left(\;Q\;\right)\leq \frac{\#\text{FP}+\#\text{TN}}{V}q\leq q. \end{array}$$

The algorithm is as follows [15]:

(1) Select a level *q*.
(2) Order all *V* original *p* values from smallest to largest. With *ℓ*_*v*_ representing the *v*th smallest *p* value, that is, *p*_*ℓ*_*v*__ = *p*_(*v*)_, the ordered *p* values are as follows:
  (15) $$\begin{array}{c} p_{\left(1\right)}\leq p_{\left(2\right)}\leq \cdots \leq p_{\left(V\right)}. \end{array}$$
(3) Define *r* such that it is the largest *v* for which *p*_(*v*)_ ≤ (*v*/*V*)*q* holds.
(4) Declare all voxels *ℓ*_1_ ⋯ *ℓ*_*r*_ to be active.

Genovese et al. [15] argue that this procedure controls the FDR under the assumption of* positive dependence*; that is, noise is Gaussian with nonnegative correlation. This assumption is reasonable given that smoothing images imposes increased dependency between neighboring voxels (and thus tests).

#### 2.2.3. Uncorrected Threshold with Minimum Cluster Size

Based on simulation studies, Lieberman and Cunningham [13] proposed a more ad hoc two-step procedure that aims for a better balance between FP and FN. In the first step, the test image is thresholded at *u*, corresponding to an uncorrected *α* of, for example, 0.005. In the second step, only those voxels belonging to a cluster with minimal cluster size of 10 are selected.

*Relation with Cluster-Based Significance Testing*. It should be noted that the method of an uncorrected threshold with a minimum cluster size shows superficial resemblances with cluster-based significance testing procedures. Cluster-based significance testing is a popular method to detect activation [16]. It is however fundamentally different in nature from the procedures described above. Indeed, it uses topological features rather than purely voxel-based characteristics and therefore relies on different assumptions.

As suggested by the reviewers, we added this method to our comparison in the simulations for completeness (see Section 3). More specifically, we added the cluster size (*S*) based significance testing with FWE-corrected and FDR-corrected *p* values. This corresponds to the two-step procedure but the minimum cluster size *S* is obtained based on cluster significance instead of fixing it at 10. Similar to the two-step procedure, a first threshold *α* is chosen and only clusters that are sufficiently large are retained as significant. Without going into technical details for both permutation-based and parametrical inference (which can be found in, e.g., [16, 35, 36]), this procedure determines the significance of a cluster in order to obtain the minimum cluster size *S*. More specifically, in a first step, after having set a sufficiently high fixed first threshold (e.g., *α* = 0.001), clusters are determined by a cluster-forming algorithm. In a second step, for each of these suprathreshold clusters, the probability to observe a cluster of size *S* under the null hypothesis of no activation can be determined. These cluster *p* values can be corrected to control either the FWE (further referred to as cluster-FWE) or the FDR (further referred to as cluster-FDR) at cluster level.

In the two-step procedure with a fixed cluster size of 10, the first threshold *α* can be varied (empirically). For cluster-based inference on the other hand, it is important to note that the null distribution of cluster sizes relies on the assumption that the first (cluster-forming) threshold remains fixed at a stringent *α*-level, typically of *α* = 0.001. This implies that, in the simulations, it is the minimum cluster size *S* that is varied empirically for the cluster-based approach (by imposing different statistical thresholds for cluster sizes through varying the FWE or FDR) and not the cluster-forming threshold *α*.

### 2.3. Inference

#### 2.3.1. Parametric Inference

If one is willing to make distributional assumptions for the test statistic of interest, one can easily derive the thresholds for inferential decision making. We first discuss such parametric inference for the FWE and next for the FDR and the two-step approach.

For the FWE correction, one can rely on Random Field Theory (RFT) to derive the null distribution of max⁡(*T*_*v*_). Using two essential approximations from* Gaussian* Random Field Theory (which we will not discuss in full detail here, more details can be found elsewhere, e.g., [8, 34]), we have that

(16) FWE=Pmax⁡Tv>u ∣ global  H0

(17) ≈Pχu>0

(18) ≈Eχu.

In expression (17), the FWE is approximated by the probability that the Euler Characteristic *χ*_*u*_ is larger than 0. *χ*_*u*_ basically counts the number of clusters under the null hypothesis, that is, a collection of neighboring voxels for which *T*_*v*_ > *u* holds. If the cluster-forming threshold *u* is set sufficiently high the probability to observe more than 1 cluster is neglected and one can approximate the FWE with expression (18). The expected value of *χ*_*u*_ is estimated through a closed-form approximation that uses information about the smoothness of the image of test statistic [8, 34]. Not only does the method take into account the spatial character of the data through the smoothness, but also its computational efficiency is a major advantage [9]. It is challenging however to satisfy the main underlying assumptions needed for valid inference, that is, normally distributed noise, sufficient smoothing, and a sufficiently high threshold (see, e.g., [34, 37]).

For the FDR corrected inference and the two-step procedure, uncorrected *p* values that are based on the usual *t* distributions of the test statistics which rely on normally distributed noise, as obtained from the OLS and WLS approach, can simply be used.

#### 2.3.2. Permutation-Based Inference

Although some tools exist to verify the distributional assumptions underlying the test statistic (e.g., [38]), there is no widespread tradition to check those assumptions in fMRI data analysis [39]. The parametric null distributions indeed often rely on strong assumptions, which are seldom entirely fulfilled [10]. Therefore one could alternatively use nonparametric approaches such as bootstrap (e.g., [40–42]) and permutation procedures (e.g., [11, 43, 44]). Using resampling techniques, the permutation approach, for example, guarantees (asymptotically) valid inference at nominal levels by creating a null distribution conditional on the observed data, but that advantage comes at the cost of increased computational effort.

Focusing on second-level analysis and the scenario where one simply wants to test for activation over all individuals (i.e., the design matrix **X**_**M**_ is a vector of 1's), permutation-based testing proceeds as follows:

(1) Define *P*, the number of permutations; the higher *P*, the higher the precision of the empirical null distribution. However, the computational burden also increases with increasing *P*.
(2) Compute for each voxel *v* the test statistic in the original sample: *T*_*v*0_ for each voxel.
(3) Create *P* new samples by randomly flipping the sign of some of the elements in **X**_**M**_; that is, for randomly chosen individuals the 1 is changed into −1 [10] (if the individuals belong to different groups or the study design is more complex, more appropriate schemes can be found in, e.g., [45]).
(4) For each of the *P* (with *p* = 1,…, *P*) samples compute the test statistic *T*_*vp*_.
(5) The permutation null distribution for voxel *v* is then defined as the empirical distribution of *T*_*vp*_'s. Clearly, the smaller the number of permutations *P* is, the more discrete the null distribution will be.

Within a mass-univariate approach, empirical *p* values are obtained per voxel using *P* (*T*_*pv*_ ≥ *T*_*v*0_), the probability to observe a test statistic in the permutation null distribution that is at least as large as the test statistic observed in the sample at hand. The FDR correction and the two-step procedure are performed on these *p* values.

For the FWE correction, permutation based inference proceeds via the empirical sampling of the maximum statistic over all voxels to obtain the null distribution of the maximum statistic. This implies that in step (4) the maximum over the test statistic of all voxels is calculated: *T*_*p*_ = max⁡(*T*_*pv*_) with (*v* = 1,…, *V*).

## 3. Simulations

### 3.1. Data Generation

For every subject (*m* = 1,…, 15) and for every voxel in a 3-dimensional space (45 × 45 × 45), we generate a time series **y** for the signal on the first level using the following model:

(19) $$\begin{array}{c} y=X\beta +Zd+\epsilon , \end{array}$$

with **β** = [*β*_0_, *β*_1_]^*t*^ and with **X** the design matrix, consisting of a column for the intercept and a column describing the expected signal under a simple block design. **Z** is identical to **X**, and **d** contains a random intercept *d*_0_ and random slope *d*_1_. The random intercept variance was set to zero, while a random slope *d*_1_ is drawn from *N*(0, *σ*_*d*_1__^2^) for every subject to allow for heterogeneous effects of **X** on **y** between subjects. For every subject, voxel, and time point, **ϵ** is drawn from *N*(0, *σ*_*m*_^2^). In the simulation study no temporal correlation was induced as this unnecessarily might influence our variance estimates and consequent inference (see, e.g., [46], for an investigation of the impact of modeling the temporal autocorrelation in fMRI). We further define a signal-to-noise ratio (SNR) as the maximum amplitude (**x** *β*_1_) divided by *σ*_*d*_1__ and focus on a simple contrast **c** **β** with **c** = [0,1].

The between-subjects standard deviation, *σ*_*d*_1__, was set such that SNR = 1 (low signal strength) or SNR = 2.5. The variance *σ*_*m*_^2^ is either constant or varying over the *M* subjects. To ensure comparability between both scenarios in terms of the average total amount of variability, the variance *σ*_*m*_^2^ under the constant scenario is set to the average of all values under the varying scenario.

We use the neuRosim R package [47] and a canonical HRF to set up the first level activation [26] in (19). In total there are 1934 active voxels, distributed over two clusters, and 89191 inactive voxels in a 45 × 45 × 45 volume (±2.5% of the voxels). The noise images that were added to the activation image were minimally smoothed in order to comply with the basic assumptions for RFT [3, 34, 39].

In total, 1000 simulations are performed for all 4 data generating mechanisms (2 SNR and constant versus varying *σ*_*m*_^2^).

### 3.2. Analysis and Evaluation Details

#### 3.2.1. Analysis

We focus on the OLS and WLS approach to combining the individual evidence from the *M* subjects. FSL (version 5.0.7, [29]), one of the most frequently used software packages to analyze fMRI data [5], has both methods implemented. First, the estimates $c\hat{\beta }$ (see (1)) are obtained and next used for the second-level analysis. In the WLS approach, for every subject *mσ*_*m*_^2^ is estimated (see (6)) and then used to weight the evidence per subject as outlined in (11). For the parametrical inference in the OLS case, inference is based on the *t* distribution with *M* − 1 degrees of freedom. The WLS method uses an intrinsic Bayesian procedure that takes into account both the subject-specific variability and the variability on the estimation of **c** **β**. Further inference proceeds via a back-transformation of the posterior probability *P* (**c** **γ** > 0∣**b**) (see (3) and [7]) to a *Z*-map.

For both the OLS and the WLS we use the permutation technique based on* sign-flipping*; see Section 2.3.2. The command line tool randomised allows for permutation based on the OLS method. For the WLS approach we followed the same protocol, but via an in-house R script with the test statistic as in (9). The permutation null distributions are based on 5000 permutations. On a standard laptop computer the computational time for the OLS permutation was less than 10 minutes compared to over about 40 minutes for the WLS permutation. We note that compared to the FSL implementation our in-house script was not fully optimized to speed up computational time.

#### 3.2.2. Evaluation

The performance of the different combination of techniques is evaluated based on the Receiving Operating Characteristics (ROC) curves. The ROC curves show the true positives (TP) rate in function of the false positives (FP) rate, with the FPs defined as voxels that are declared active but not in the true activation region and the TPs as the voxels that are declared active and in the true activation region.

ROC-curves provide a means to investigate the balance between the FP and TP rate; however, bias may be introduced for imbalanced data. As in fMRI, there are typically more true inactive than true active voxels; we also provide the Matthews correlation coefficient [48]. This measure takes into account the four cells as displayed in Table 1 and is therefore a more comprehensive measure for the quality of a test criterion, even for imbalanced data (see, e.g., [49], for an application in the genetical context). The Matthews correlation coefficient (MCC) is calculated as follows:

(20) MCC=TP×TN−FP×FNTP+FPTP+FNTN+FPTN+FN.

Values close to 1 indicate more correct decisions, values close to 0 indicate random decisions, and values close to −1 indicate more incorrect decisions.

Furthermore we study stability through the variation on the number of correctly selected voxels. Stable methods are methods that do not induce much variability on the number of selected voxels. At last, from the above, it should be clear that all measures are defined in voxel-based way.

### 3.3. Results

**Figure 1:**
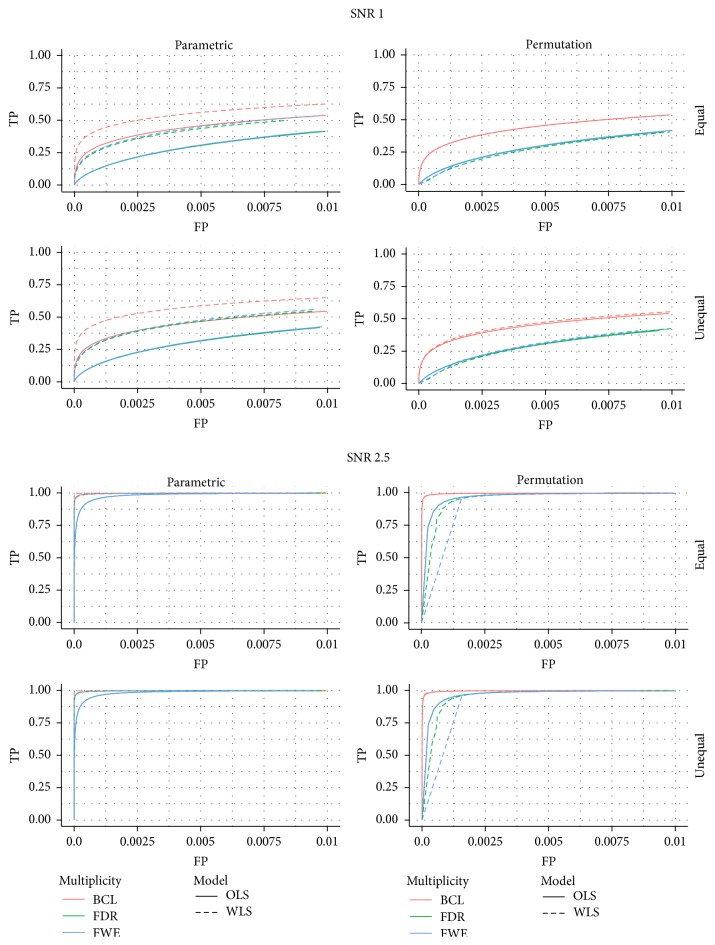
ROC for the low signal strength (SNR = 1) and for the high signal strength (SNR = 2.5); for differences in the subject-specific variability (*unequal*) or identical subject-specific variability (*equal*); for permutation-based inference and for parametric inference. FWE: familywise error correction, FDR: False Discovery Rate correction, and BCL: two-step procedure with a Bonferroni-like first threshold and minimal cluster size of 10. OLS: Ordinary Least Squares approach and WLS: Weighted Least Squares approach.

In Figure 1 we present the ROC curves under each of the four data generating mechanisms (low versus high SNR in left versus right panel, equal versus unequal *σ*_*m*_^2^ in the upper versus lower panel). In total 12 ROC curves are presented, one for each of the 2 × 2 × 3 combinations of selection procedures (OLS versus WLS, parametric versus nonparametric inference, FWE versus FDR versus 2-step procedure). We summarize the most important findings below.

First, we find that under all scenarios the two-step procedure with a Bonferroni-like first threshold and minimal cluster size of 10 (further denoted as BCL) has a better trade-off between FP and TP than the FWE-control or FDR-control.

Second, under both high and low signal strength, the ROC of the permutation-based method and the parametric inference have very similar shapes at almost the same height when focusing on the OLS approach. When considering the WLS approach, one finds that the ROC curves are substantially higher with permutation-based inference than with the parametric inference under both SNR (regardless of the type of control).

Third, in almost all panels of Figure 1 we find a good performance of the WLS versus the OLS method under the parametric approach, regardless of the type of multiplicity control. When permutation-based inference is used a similar performance of OLS and WLS is observed when the SNR is low, but the WLS seems to perform worse than OLS when the SNR is high. It should be noted that this is due to the discreteness of the permutation-based inference, which is mostly apparent when the signal is strong.

**Figure 2:**
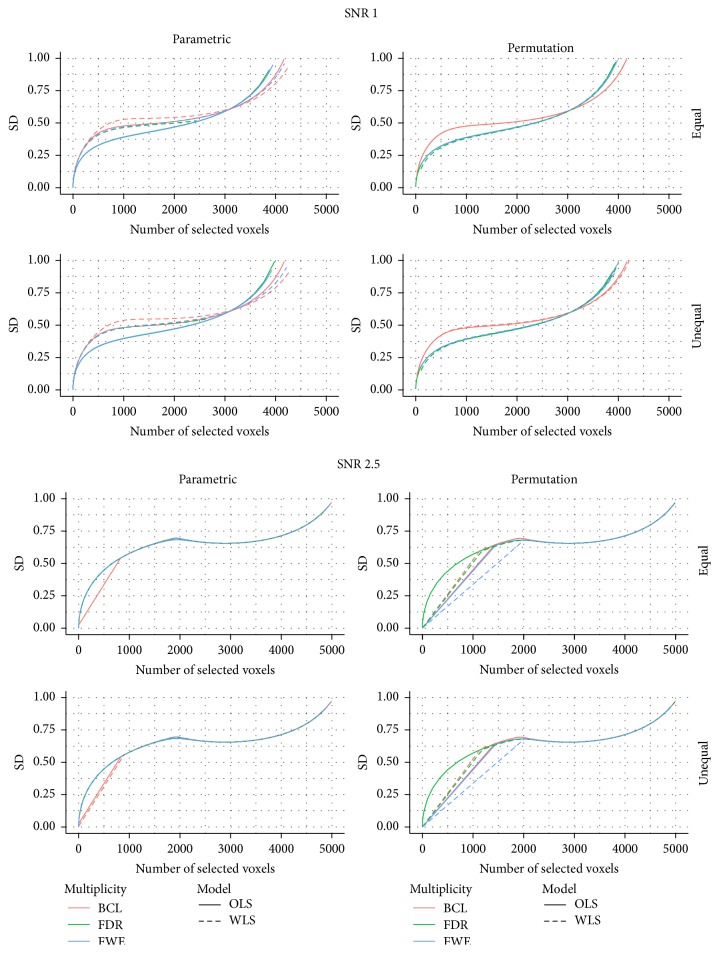
Matthews correlation coefficient (MCC) for the low signal strength (SNR = 1) and for the high signal strength (SNR = 2.5); for differences in the subject-specific variability (*unequal*) or identical subject-specific variability (*equal*); for permutation-based inference and for parametric inference. FWE: familywise error correction, FDR: False Discovery Rate correction, BCL: two-step procedure with a Bonferroni-like first threshold and minimal cluster size of 10, and OLS: Ordinary Least Squares approach and WLS: Weighted Least Squares approach.

In Figure 2, the MCC is depicted for, respectively, a low and high signal strength with respect to the total number of selected voxels (FP + FN). While the findings based on the pattern of the ROC-curve are mostly confirmed in these figures, the differences under high SNR are somewhat less pronounced. This may indicate that under high SNR the decisions diverge less than when the SNR is lower for a same number of selected voxels.

**Figure 3:**
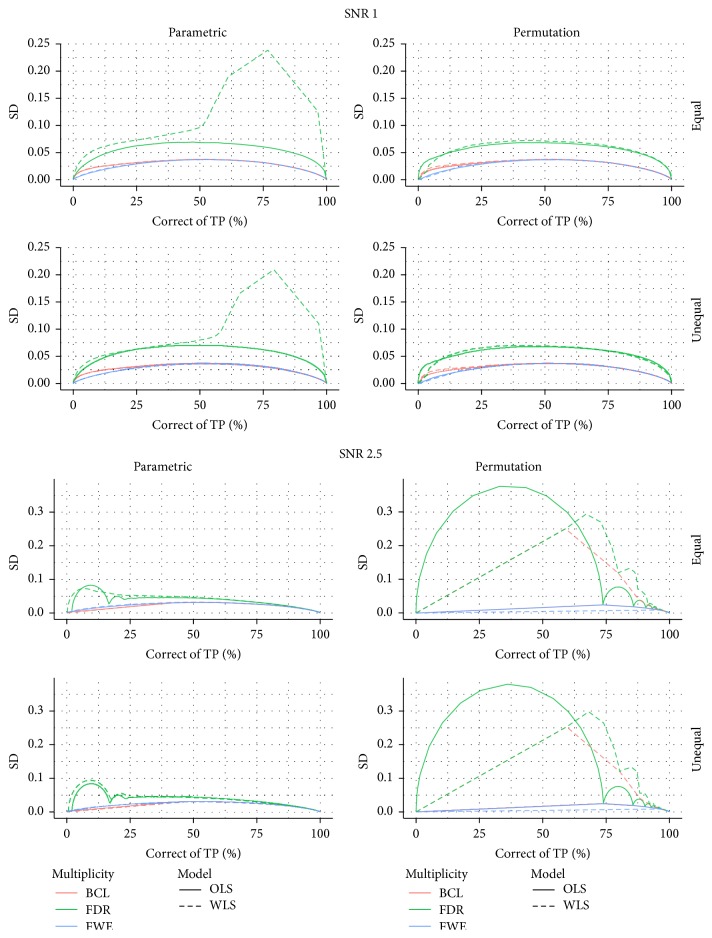
Stability plot for the number of correctly selected voxels in the simulation with low signal strength (SNR = 1) and for the high signal strength (SNR = 2.5); for differences in subject-specific variability (*unequal*) or identical subject-specific variability (*equal*); and for permutation-based inference and for parametric inference. FWE: familywise error correction, FDR: False Discovery Rate correction, and BCL: two-step procedure with a Bonferroni-like first threshold and minimal cluster size of 10. OLS: Ordinary Least Squares approach and WLS: Weighted Least Squares approach.

Figure 3 shows the proportion of correctly selected voxels on the *x*-axis and its corresponding standard deviation on the *y*-axis. For all 4 data-generating mechanisms, we find that the FDR correction for multiple testing results in more variability than the other two procedures that correct for multiple testing. We also find that the FWE correction results in slightly more variable results than the BCL based corrections. Furthermore, this pattern is not altered by the choice for permutation-based inference or parametric inference. One exception is however observed. Indeed, we find that, for the WLS procedure, under the high SNR, the BCL procedure becomes more variable than the FWE procedure. We attribute this, again, to the discreteness of the permutation method and the high signal present in this simulation.

**Figure 4:**
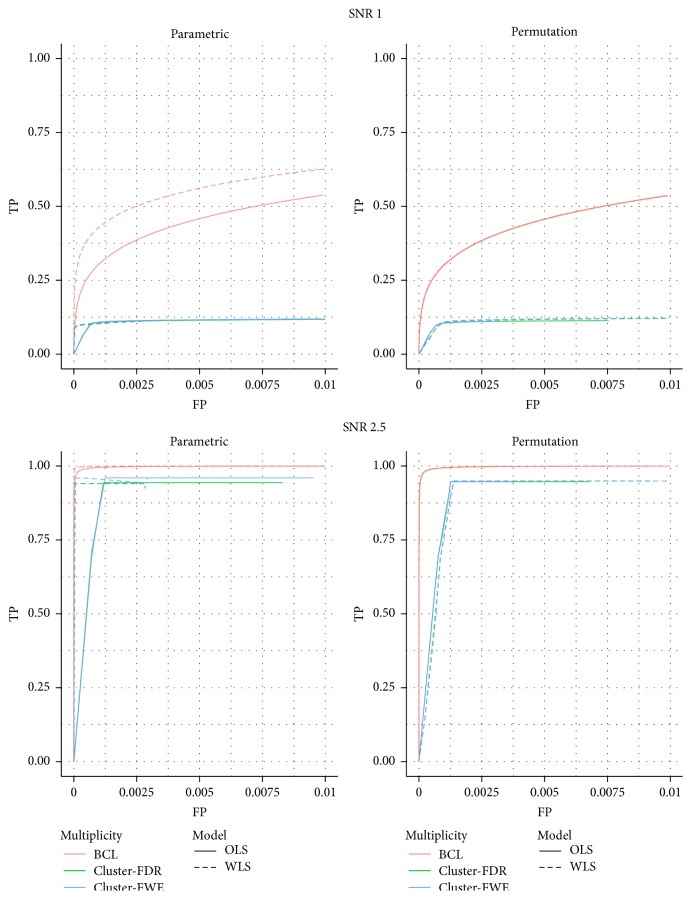
ROC for the low signal strength (SNR = 1) and for the high signal strength (SNR = 2.5) with identical subject-specific variability; for permutation-based inference and for parametric inference for cluster-based inference with *α* = 0.001: cluster-FWE: familywise error correction based on cluster-size inference, cluster-FDR: False Discovery Rate correction based on cluster-size inference, and BCL: two-step procedure with a Bonferroni-like first threshold and minimal cluster size of 10. OLS: Ordinary Least Squares approach and WLS: Weighted Least Squares approach.

Figure 4 depicts the comparison between the BCL procedure and the pure cluster-size based inference in the ROC-curve in the simulations with no between-subject differences in the residual variability. The results for the case* with* differences in the within-subject variability and the results for the stability plots and the MCC are presented in Appendix B. We note that, due to the first fixed threshold in pure cluster-based testing, the maximum number of selected voxels is limited. For the ROC-curves and for the stability we find discrete patterns. These are a logical consequence of our simulation setup, in which two relatively large clusters are set active. Based on the ROC-curve we find a good trade-off between FP and TP for the cluster-based inference when the SNR is high, but not when the SNR is low. For the stability, it is hard to draw conclusions based on the observed results due to the above-mentioned limitations.

**Figure 5:**
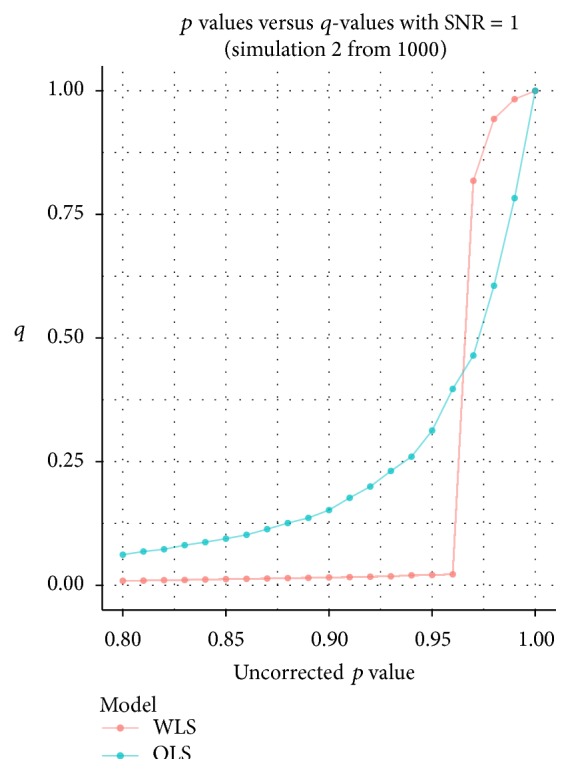
Uncorrected *p* values for the OLS and the WLS procedure, with their corresponding FDR corrected *q*-values based on one specific simulation under SNR = 1 with equal variance among subjects.

Finally note that, under the lowest signal strength, we find a peak in the variability for the WLS approach in combination with the FDR correction. Further inspection of the *p* values for the WLS approach reveals that this is due to more discreteness in the highest *p* values compared to the OLS procedure (Figure 5).

## 4. Real Data Example

### 4.1. Human Connectome Project Dataset

To check the findings from the simulation study on real data, we use data from the Human Connectome Project (HCP, [50]). Those data are analyzed on the first level, using a standard protocol that is described elsewhere [51]. To mimic a typical fMRI study with about 15 subjects, we select the first 15 subjects (subject identifiers can be found in Appendix A.) from the HCP dataset with a focus on contrast 4, which entails the difference between a mathematical task and a story-telling task.

### 4.2. Stability of the Selected Voxels

For the HCP data, we determine the stability of the different proposed methods by bootstrapping subjects from the original sample, that is, drawing subjects with replacement from the original sample. In total, 100 bootstrap samples are taken. The number of active voxels at level 2 is determined in each of these bootstrapped datasets, using one of 12 the aforementioned combinations for inference at the second level. The stability on the number of selected voxels over bootstrap samples is further assessed by considering the* reselection rate* of a specific voxel, which is the proportion of bootstrap samples in which that voxel is declared active.

### 4.3. Results

**Figure 6:**
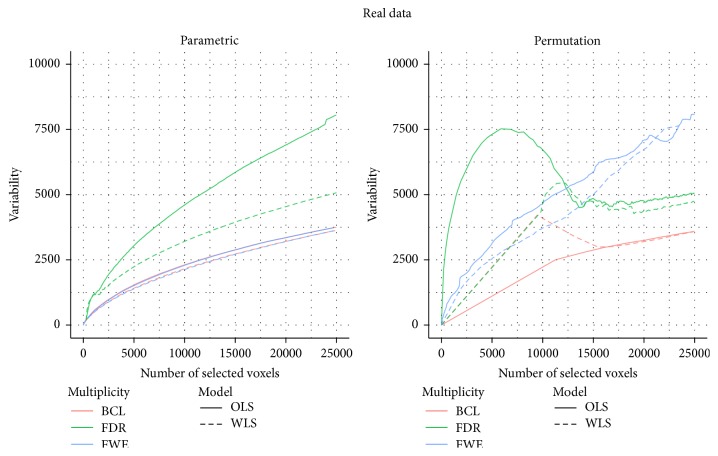
Stability plot for the number of selected voxels for *n* = 15 of the HPC dataset for permutation-based inference and for parametric inference. FWE: familywise error correction, FDR: False Discovery Rate correction, and BCL: two-step procedure with a Bonferroni-like first threshold and minimal cluster size of 10. OLS: Ordinary Least Squares approach and WLS: Weighted Least Squares approach.

In Figure 6, we find the same pattern as in the simulations when using parametric inference, that is, the FDR based correction for multiple testing results in more variability on the number of selected voxels. Also, we find that the FWE and the BCL correction result in similar variability. This finding holds for both the WLS and the OLS approach. In contrast to the simulation study, we find however that the WLS approach is always less variable than the OLS approach for a given type of multiplicity control.

For the permutation-based inference we find that when the number of selected voxels is relatively low (less than ±5% of the ±200.000 voxels) the FDR correction with the OLS is far more variable than all other combinations. We note again that the WLS suffers from the discreteness of *p* values in the permutation-based inference when the FDR correction is used. Due to this discreteness, several small original *p* values are converted to only one corrected *q* value, causing the straight line from the origin to the first point. For the two-step procedure, there is a similar artifact when using WLS. This can be attributed to the fact that the lower *p* values do not occur in clusters larger than 10, until these reach a certain threshold that results in a huge amount of activation. If more than 5% of the voxels are selected, the results are more variable if one uses the FWE correction for multiple testing, compared to the other methods.

**Figure 7:**
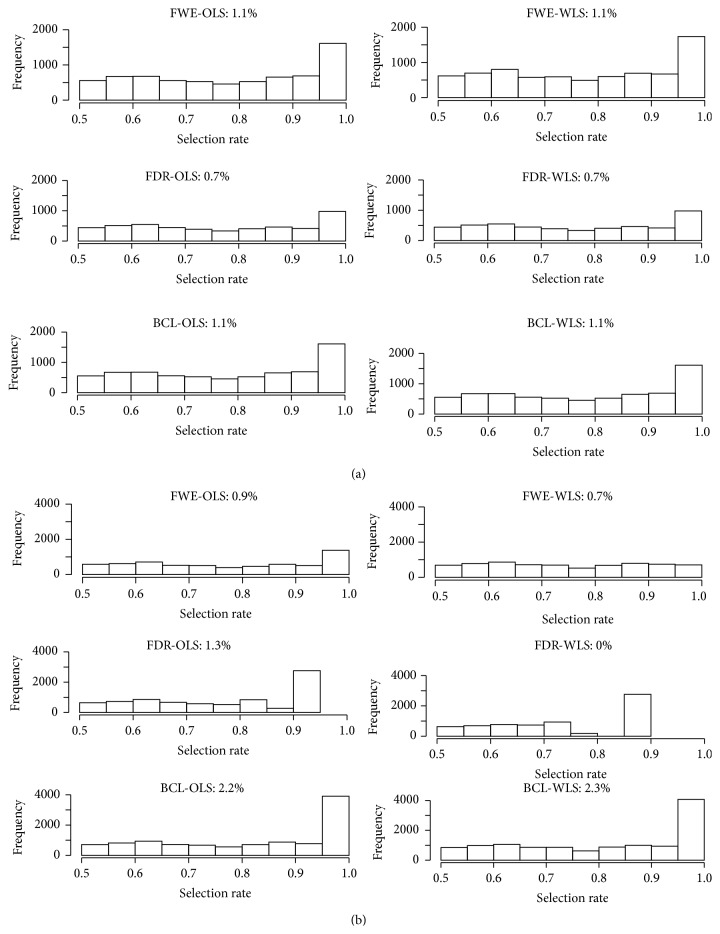
Plot with the reselection rates of the voxels that are larger than 0.5 over 100 bootstrap samples for real data for parametric inference (a) and for permutation-based inference (b). FWE: familywise error correction, FDR: False Discovery Rate correction, and BCL: two-step procedure with a Bonferroni-like first threshold and minimal cluster size of 10. OLS: Ordinary Least Squares approach and WLS: Weighted Least Squares approach. The indicated percentage denotes the number of voxels that is declared active in more than 90% of the bootstrap cases.

Based on Figure 6, we next determine the thresholds for which 10.000 voxels are selected on average over the 100 bootstrap samples. These thresholds are then used to determine the reselection rate of each specific voxel over the 100 bootstrap samples.  Figure 7 depicts the histograms of the reselection rates that are larger than 50%. The header of each histogram shows the percentage of voxels that are selected in more than 90% of the samples.

From Figure 7 we find the highest reselection rates when using the FWE or BCL multiplicity control in the parametric inference framework (i.e., the 6 upper panel histograms). In the permutation-based inference framework (i.e., the 6 lower panel histograms), we find that the FDR achieves higher reselection rates than the FWE if the OLS approach is used, but the highest reselection rates are found with the BCL multiplicity control with both the OLS and the WLS approach.

**Figure 8:**
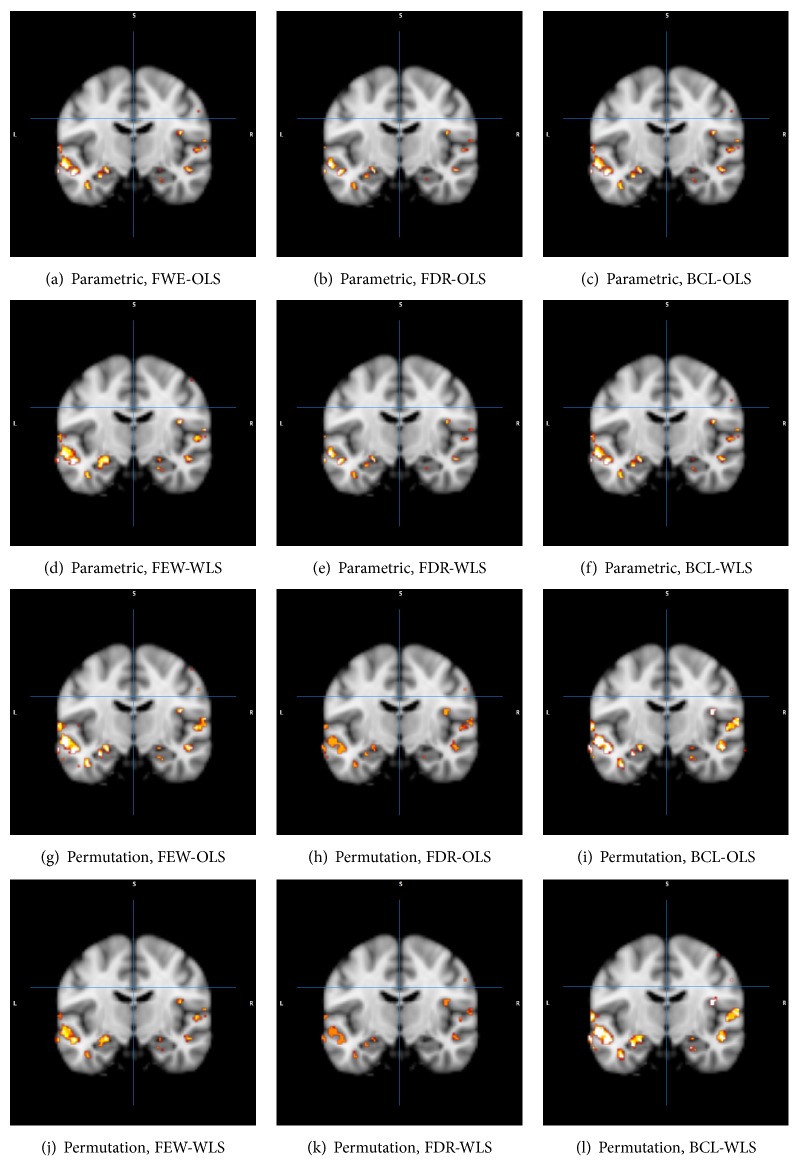
Plot with the reselection rates that are larger than 0.75 for the HPC data for parametric inference (a–f) and for permutation-based inference (g–l). FWE: familywise error correction, FDR: False Discovery Rate correction, and BCL: Bonferroni-like first threshold and minimal cluster size. OLS: Ordinary Least Squares approach and WLS: Weighted Least Squares approach. The average number of activated voxels was kept constant for all cases. Red/orange: closer to 0.75; white: closer to 1.

To take into account the localization of voxels that are frequently reselected, we also constructed brain images in Figure 8, where we identified all voxels that have a reselection rate of at least 75%. Although we acknowledge that the slice depicted is only exemplary, the above-described trends are clearly confirmed.

### 4.4. Test-Retest Correspondence

**Figure 9:**
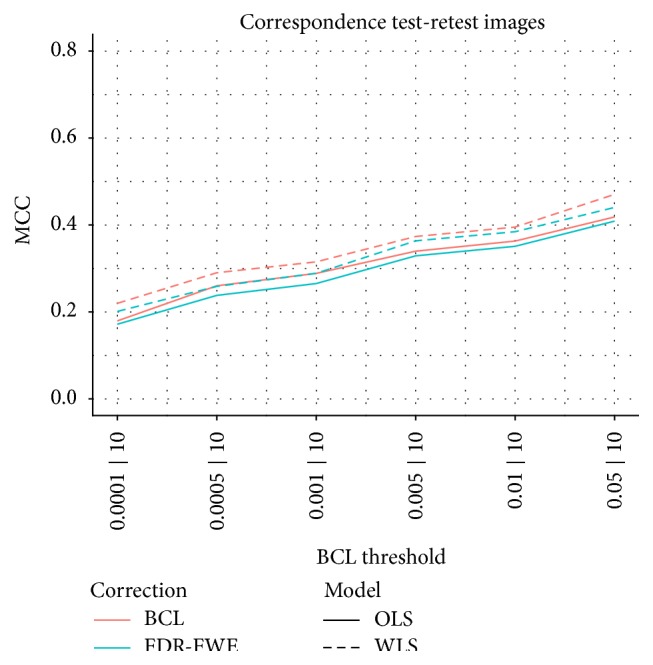
Test-retest correspondence measured trough the correspondence between two binary images (selected/nonselected voxels). Each BCL threshold corresponds to a specific number of selected voxels which may vary between images but not between methods.

As suggested by one of the reviewers, stable methods should reflect more similar results using different real samples. To study this, we used an additional run for each of the 15 subjects in the HCP data. We exemplary demonstrate this test-retest similarity for the parametrical analysis. We matched the number of selected voxels per image in the FWE/FDR method by the respective numbers that are found using the two-step BCL procedure. Indeed, when selecting the *N* voxels with the *N* smallest *p* values, the FWE and FDR method results are identical. This matching on the number of selected voxels is motivated by the simulation findings that the larger number of selected voxels results in a higher MCC. In a test-retest setting, the MCC coincides with the correlation between two binary images (selected/nonselected voxels). In Figure 9 we see that indeed the BCL outperforms the FDR/FWE and that the WLS outperforms the OLS. We note however that this methods has a major drawback as it does not allow us to calculate the variability on these numbers and it requires a second sample.

## 5. Discussion

In this study we investigated both the balance between true positives (TP), true negatives (TN), false positives (FP), and false negatives (FN) and data-analytical stability of methodological choices in the second-level analysis of fMRI data. Following the traditional evaluation of techniques in the fMRI literature, we first focused on the balance between FP and TP, using ROC-curves, and on the Matthews correlation coefficient (MCC), a measure that takes all possible decisions into account. Aiming for more reproducible brain imaging research, we believe however that data-analytical stability is also an important criterion that offers an additional unique perspective on the behavior of methods. While studies using the criterion of data-analytical stability are sparse and mostly focused on the first-level inferential decisions (e.g., [4, 52], for, resp., a focus on mass univariate inference and topological inference), this study filled this gap through considering data-analytical stability of different methods at the second-level analysis. Unlike the NPAIRS framework [53, 54] that allows exploring overall stability, we furthermore focused on the* selected* voxels, obtained via thresholded images, when assessing the data-analytical stability.

More specifically, we assessed in this paper the impact of three different choices that the researcher has to make when analyzing fMRI data at the second level: (1) should one use a WLS-approach or an OLS-approach, (2) should one rely on parametric assumptions for the test statistic or rely on a nonparametric framework, such as permutation-based inference, and (3) which type of control should one use to limit the multiplicity issue. The impact of these choices was assessed from the ROC-curves, MCC, and the data-analytical stability perspective.

For the balance in the decision context, based on the ROC-curves and the MCC, results were pretty clear when parametric inference is used. Regardless of the choice of the multiple testing correction, we found that the WLS-method yields a better balance between FP and TP than when the OLS-method is used. While the MCCs confirmed most of the results based on the ROC-curves, they revealed the fact that differences are more obvious when the SNR was low. Under the high signal strength, the balance in the decision context did not diverge remarkably between methods. These findings on the balance between FP and TP are in line with Mumford and Nichols [18], although the magnitude of the difference between WLS and OLS was more pronounced, based on the ROC-curves, in our simulation study. When permutation-based inference is used, there were barely any differences between OLS and WLS. We found however that there were some effects of discreteness when permutation-based inference was used in combination with WLS. In the simulation settings this was associated with spiky patterns under a high SNR due to substantial jumps in the number of voxels that are selected. But also in the real data application, we found some evidence for discreteness with the WLS statistic when jumps in the activation occur. When comparing the parametric with the nonparametric approach, we found in contrast to Thirion et al. [43] no evidence for a better performance of permutation based inference. Note however that in all our simulation settings the basic assumptions of parametric inference were satisfied (Gaussian noise and sufficient smoothing). Upon inspection of the ROC-curves we also found in our simulation study that the two-step procedure, which ignores multiplicity first but requires a minimal cluster size next, outperforms the traditional FWE-control and FDR-control.

From a data-analytical stability perspective, there were substantial differences between the three approaches we considered for multiple testing correction. In line with previous findings at the first level of analysis [21, 52], FDR-based corrections for multiple testing resulted in more variable selections. In both the simulation study and the real data application, we found that FWE based correction for multiple testing and a two-step procedure result in more stable results, as assessed by the variability on the number of selected voxels. This weaker performance of the FDR is observed, regardless of the WLS-approach versus OLS-approach, or the parametric versus nonparametric framework for inference. Interestingly, when we focused on the reselection rate of a specific voxel in the data application, we also found superior performance of the two-step procedure. As noted by one of the reviewers, the increased stability for the FWE and two-step procedures relying on parametrical inference might be attributed to the fact that these approaches exploit topological features of the data in contrast to the FDR.

While voxel-based inference is only one approach to controlling for multiple testing, several alternatives exist. Cluster-based inference (see, e.g., [35, 36]) is a very popular alternative that relies explicitly on topological features such as the cluster size and has been advocated because of the potential increase in power. However, Woo et al. [16] showed that the commonly used two-step procedure for cluster-based inference is nonrobust when too liberal first thresholds are used at the voxel level and that this results in unpractically large clusters when studies are sufficiently powered. This complicates the interpretation of the results as clusters could become as large as half of the hemisphere. In the same vein, Woo et al. [16] and Nichols [9] argue that the conceptual definition of a “significant cluster” is complicated by the fact that it is a randomly sized collection of voxels of which one can only claim that at least some are significant. We concur with Nichols [9] and Woo et al. [16] that voxel-wise inference remains a useful alternative and therefore opted for an extensive evaluation of commonly used voxel-based inference techniques.

The FP rates are evaluated only in a simulation study. While this might lack biological validity, this procedure allows us to have strict control on the ground truth and consequent determination of TN and TP. With an exhaustive simulation study (2 SNR and varying within-subject variability assumptions), we have covered some of the properties present in real data. Any simulation study comes naturally with the arbitrariness of these settings. However, compared to using real data to determine FP rates, simulation studies have the advantage to exclude unnecessary artifacts in the procedure to determine the TP and the TN (see, e.g., [55], for differences in test errors based on the design) or its underlying assumptions.

Gathering all the above-described evidence, we would recommend the brain imaging researcher to use WLS at the second level in combination with the two-step procedure, hereby relying on the parametric framework for inference. Note that throughout the paper, we have assumed that all images at the first level are correctly normalized such that individuals are perfectly coregistered. It should be stressed that further exploration of the robustness against violations of the parametric assumptions is warranted. However, the proposed strategy in this paper to assess data-analytical stability of different methods on real data could be used in any future application and ultimately reveal the best choice from a data-analytical stability perspective in practice. Such validation on real data may also yield further insight into the appropriateness of the rather ad hoc but commonly used BCL-approach which lacks inferential justification.

## Figures and Tables

**Figure 10:**
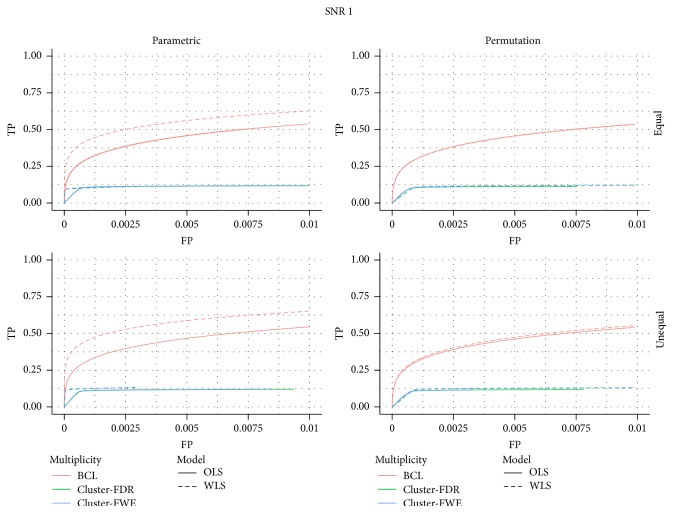
Receiver operating curve for a signal-to-noise ratio of 1 over the range [0; 0.01].

**Figure 11:**
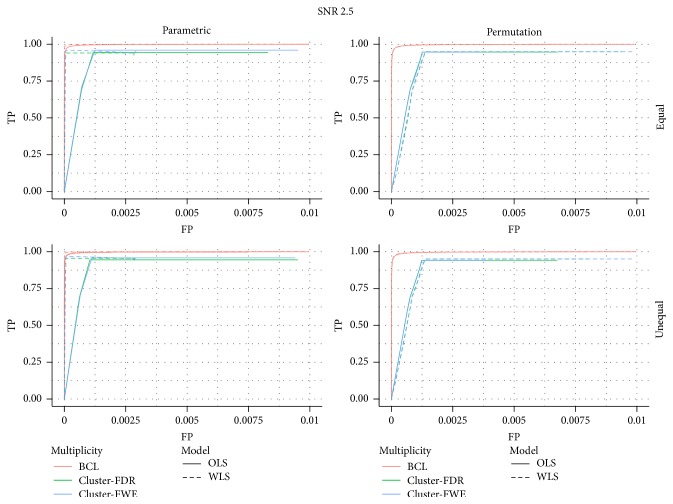
Receiver operating curve for a signal-to-noise ratio of 2.5 over the range [0; 0.01].

**Figure 12:**
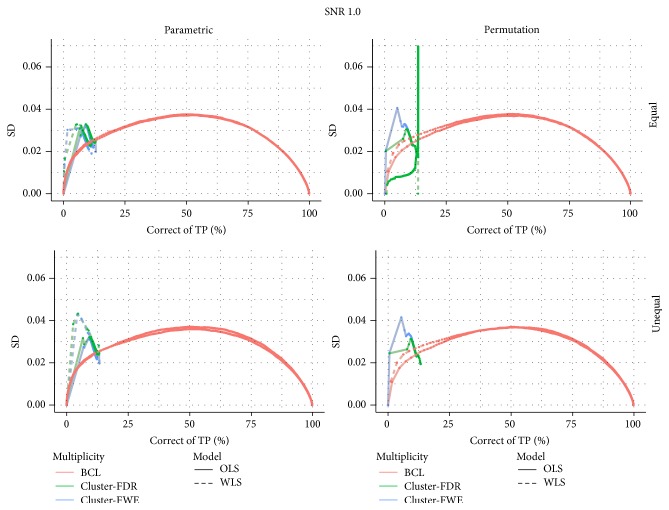
% of correctly activated voxels with their standard deviation for a signal-to-noise ratio of 1.

**Figure 13:**
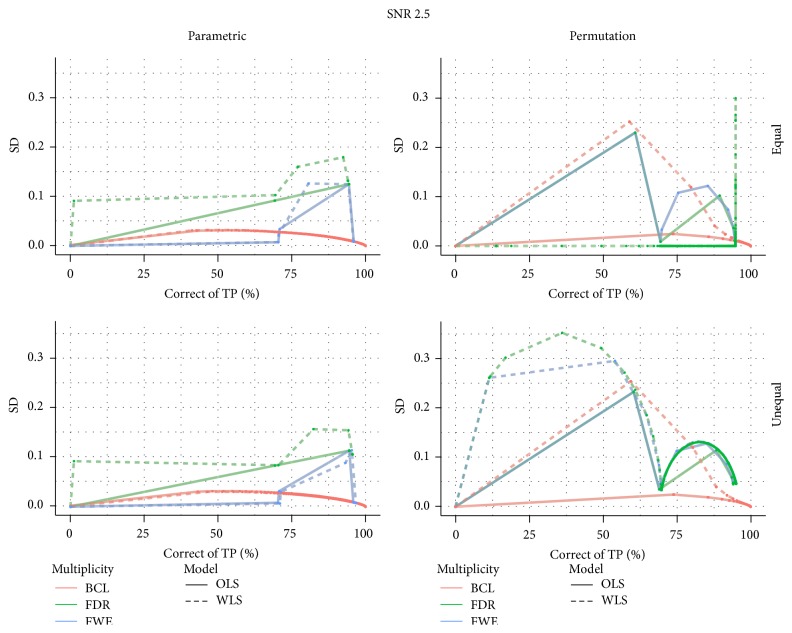
% of correctly activated voxels with their standard deviation for a signal-to-noise ratio of 2.5.

**Figure 14:**
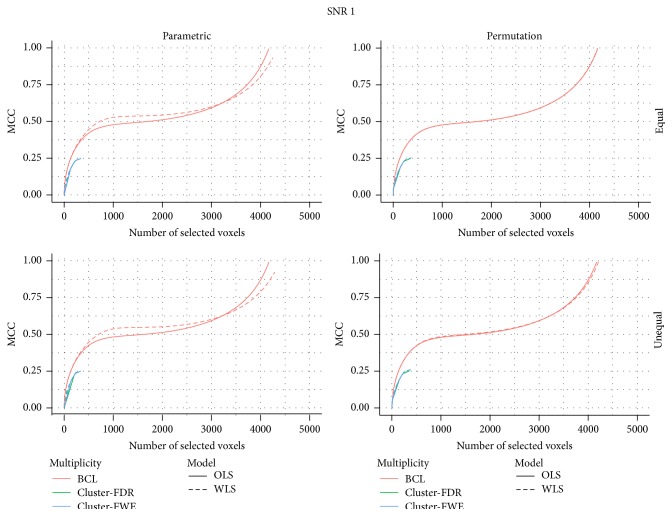
MCC for a signal-to-noise ratio of 1.

**Figure 15:**
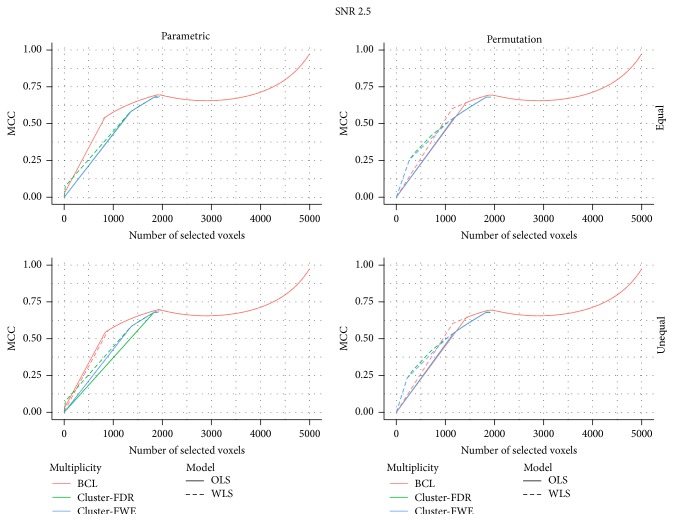
MCC for a signal-to-noise ratio of 2.5.

**Table 1:** Table of events for Null Hypothesis Significance Testing (NHST) in which evidence against a null hypothesis *H*_0_ is evaluated in the direction of an alternative hypothesis *H*_1_.

		Decision	
		Conclude *H*_0_	Conclude *H*_1_
Voxel	Active	False negative (FN)	*True positive* (TP)
	Inactive	*True negative* (TN)	False positive (FP)

## Appendices

### A. Additional Details HCP Dataset

Data were provided by the Human Connectome Project, WU-Minn Consortium (Principal Investigators: David Van Essen and Kamil Ugurbil; 1U54MH091657) funded by the 16 NIH Institutes and Centers that support the NIH Blueprint for Neuroscience Research and by the McDonnell Center for Systems Neuroscience at Washington University.

The list of subject identifiers used in the real data application of this study can be found in

(A.1) $$\begin{array}{c} \begin{array}{ccc} 100408 & 101915 & 103414\\ 105115 & 106016 & 110411\\ 111312 & 111716 & 113619\\ 115320 & 117122 & 118730\\ 118932 & 120111 & 122317 \end{array} \end{array}$$

Subjects come from the 80 unrelated subjects dataset, release *Q*3 [50].

### B. Additional Figures on the Relationship between Cluster-Based Inference and the Uncorrected Threshold Method with Minimal Cluster Size

This section contains the additional figures in which the BCL procedures are compared with cluster-based inference procedures. For all of the following pictures the following abbreviations are used: (1) SNR = 1: low signal strength, SNR = 2.5: high signal strength; (2) cluster-FWE: familywise error correction based on cluster-size inference, cluster-FDR: False Discovery Rate correction based on cluster-size inference, and BCL: two-step procedure with a Bonferroni-like first threshold and minimal cluster size of 10; (3) OLS: Ordinary Least Squares approach and WLS: Weighted Least Squares approach; (4) unequal: differences in the subject-specific variability, equal: identical subject-specific variability.

#### B.1. ROC-Curves

In Figures 10 and 11 the voxel-based ROC-curves are depicted.

#### B.2. Stability on the Percentage of TPs

In Figures 12 and 13 the voxel-based stability plots are depicted.

#### B.3. MCC

In Figures 14 and 15 the voxel-based stability plots are depicted.
